# Approach for enhancing the accuracy of semantic segmentation of chest X-ray images by edge detection and deep learning integration

**DOI:** 10.3389/frai.2025.1522730

**Published:** 2025-04-16

**Authors:** Lesia Mochurad

**Affiliations:** Lviv Polytechnic National University, Lviv, Ukraine

**Keywords:** semantic segmentation, medical imaging, edge detection, deep learning, U-Net model

## Abstract

**Introduction:**

Accurate segmentation of anatomical structures in chest X-ray images remains challenging, especially for regions with low contrast and overlapping structures. This limitation significantly affects the diagnosis of cardiothoracic diseases. Existing deep learning methods often struggle with preserving structural boundaries, leading to segmentation artifacts.

**Methods:**

To address these challenges, I propose a novel segmentation approach that integrates contour detection techniques with the U-net deep learning architecture. Specifically, the method employs Sobel and Scharr edge detection filters to enhance structural boundaries in chest X-ray images before segmentation. The pipeline involves pre-processing using contour detection, followed by segmentation with a U-net model trained to identify lungs, heart, and clavicles.

**Results:**

Experimental evaluation demonstrated that using edge-enhancing filters, particularly the Sobel operator, leads to a marked improvement in segmentation accuracy. For lung segmentation, the model achieved an accuracy of 99.26%, a Dice coefficient of 98.88%, and a Jaccard index of 97.54%. Heart segmentation results included 99.47% accuracy and 94.14% Jaccard index, while clavicle segmentation reached 99.79% accuracy and 89.57% Jaccard index. These results consistently outperform the baseline U-net model without edge enhancement.

**Discussion:**

The integration of contour detection methods with the U-net model significantly improves the segmentation quality of complex anatomical regions in chest X-rays. Among the tested filters, the Sobel operator proved to be the most effective in enhancing boundary information and reducing segmentation artifacts. This approach offers a promising direction for more accurate and robust computer-aided diagnosis systems in radiology.

## 1 Introduction

Segmentation of chest X-ray images, particularly of the lungs, heart, and clavicles, is a critical and challenging problem in diagnostic processes, as it allows for precise anatomical delineation, which is essential for cardiothoracic assessment and pathology detection. Despite significant advancements, medical image segmentation remains a key challenge due to the complexity of anatomical structures, variations in imaging conditions, and the presence of noise. This is key for many clinical applications, such as pathology detection, treatment planning, and disease progression monitoring (Ma et al., 2024; De Fauw et al., 2018; Berezsky et al., 2024; Honchar et al., 2025). However, despite significant advances, current segmentation methods still face several challenges, including low accuracy in distinguishing structures with similar intensity values, sensitivity to noise, and poor generalization across different datasets. These factors hinder the reliable use of automated segmentation in clinical practice.

For example, accurate segmentation allows doctors to efficiently detect abnormalities such as tumors or other changes and plan surgical interventions correctly, directly affecting the success of treatment and the overall prognosis for the patient.

The motivation for this research arises from the necessity to address these challenges. Traditional image processing methods often struggle with segmenting complex anatomical structures due to variations in contrast and shape deformations. Thresholding-based techniques, region-growing methods, and classical edge detection approaches usually fail to provide satisfactory segmentation results in complex cases. Meanwhile, deep learning-based approaches, despite their high accuracy, sometimes produce blurry boundaries or fail to capture fine structural details correctly. Research on the use of deep learning methods for medical image analysis (Litjens et al., 2017; Oliinyk et al., 2024) demonstrates that the integration of deep learning into segmentation processes is a significant breakthrough in this area. Deep neural networks, such as convolutional neural networks (CNNs) and their modifications, provide a substantial increase in accuracy and performance compared to traditional image processing methods (Mochurad et al., 2021). However, these methods alone may not always be sufficient for precise segmentation, especially when dealing with closely located anatomical structures. A major limitation of purely deep learning-based approaches is their reliance on large amounts of annotated data, which is often unavailable for rare or complex medical conditions. These technologies can adapt to complex visual structures that are often a challenge for standard approaches. Due to their ability to automatically learn from large amounts of data, they can detect subtle details and features that may be invisible to traditional algorithms.

Recent studies have shown a growing interest in deep learning methods for medical image analysis, including classification and segmentation of chest X-rays (CXR). A systematic review (Meedeniya et al., 2022) provides a comprehensive analysis of current deep-learning solutions for pneumonia and COVID-19 detection. This study highlights the importance of the availability of datasets and the latest data processing techniques to improve medical image analysis. However, it also reveals key limitations in the current literature, such as the lack of integration between classification and segmentation models, as well as difficulties in trend analysis. Furthermore, recent research suggests that hybrid approaches combining deep learning with classical image processing techniques can address some of these challenges by leveraging the strengths of both methodologies. Our work builds on these findings by addressing the problem of segmentation accuracy through a novel integration of contour detection and deep learning techniques.

A promising solution to these challenges is the integration of edge detection methods into segmentation algorithms for chest X-ray images. The integration of edge detection methods into segmentation algorithms shows significant potential for improving the accuracy of visualizing the boundaries of different structures (Liu et al., 2022). Specifically, edge detection helps to more clearly define the contours of anatomical structures, ensuring better differentiation between closely situated structures, which is particularly important for accurate medical diagnostics (Mochurad et al., 2022). Unlike purely deep learning-based approaches, edge detection can enhance the precision of segmentation by emphasizing structural boundaries. By incorporating explicit contour information, hybrid models can reduce segmentation errors in cases where deep learning-based methods alone struggle with boundary refinement. These methods can effectively enhance existing medical image segmentation techniques, offering additional tools to refine the quality and precision of analysis. When integrated with deep learning approaches, they have the potential to substantially enhance segmentation performance, minimize errors, and improve the overall accuracy of diagnostic processes.

While lung field and heart segmentation are essential for clinical diagnosis, clavicle segmentation is also of significant medical importance. Clavicles serve as key anatomical landmarks in chest radiographs and play a crucial role in trauma assessment, skeletal anomaly detection, and enhanced anatomical localization. Their segmentation aids in preventing occlusion-related artifacts in lung and heart segmentation, leading to more accurate automated analysis. Prior studies have demonstrated the importance of multi-structure segmentation in chest radiographs, particularly involving lungs, heart, and clavicles (Jafar et al., 2022; Wang et al., 2019). The accurate delineation of these structures contributes to improved cardiothoracic assessments and pathology detection (Lyu et al., 2021; Nimalsiri et al., 2023). Moreover, the proposed method integrates edge detection techniques to refine the segmentation of all three structures, demonstrating its broader applicability in medical diagnostics.

Thus, this study aims to improve the accuracy of semantic segmentation of chest X-ray images by integrating contour detection methods with the U-Net deep learning model for the segmentation of the lungs, heart, and clavicles. The novelty of our approach lies in the combination of edge detection techniques with deep learning segmentation, which enables enhanced boundary localization without the need for additional complex post-processing steps. The integration of edge detection methods with the U-Net deep learning model aims to enhance the precision of boundary delineation in medical image segmentation. Edge detection contributes to refining structural contours, which complements U-Net’s capability in semantic segmentation. This hybrid approach is motivated by the need to improve segmentation accuracy, particularly for closely situated anatomical structures that are challenging for purely deep learning-based methods.

The main contribution of the paper is as follows:

- We have conducted a detailed analysis of the current state of research in the field of medical image segmentation, particularly for CXR, focusing on deep learning approaches and U-Net-based models, and identified existing limitations and gaps.
- We propose a novel approach that integrates edge detection techniques with the U-Net deep learning model to enhance the accuracy of multi-class segmentation of anatomical structures in chest X-ray images. Unlike existing methods, our approach effectively segments both highly prominent and less distinguishable structures.
- Unlike previous segmentation methods that did not leverage edge detection techniques, we have introduced the integration of U-Net with a Sobel filter, which provided the highest accuracy and best Dice and Jaccard coefficients for segmenting the lungs, heart, and clavicles without the need for additional complex post-processing.

In addition, this study asks several key questions:

QR1: How does the integration of Sobel and Sharpe contour detection filters with the U-Net model affect the accuracy, Dice coefficient and Jaccard index in the segmentation of anatomical structures in X-ray images?

QR2: How do the results of the proposed approach (U-Net + Sobel filter) compare with other medical image segmentation methods in terms of accuracy, Dice coefficient and Jaccard index for segmentation of lungs, heart and clavicles?

QR3: What are the challenges in segmenting more complex anatomical regions such as clavicles and how can the proposed method be optimized to achieve better results in such cases?This research focuses on a deeper understanding of the impact of integrating contour detection filters on segmentation quality in the context of medical images, as well as comparing the proposed method with other state-of-the-art approaches. The results of this work can significantly contribute to the improvement of segmentation algorithms and their application in medical diagnostics, in particular for complex anatomical structures.

Here is how the manuscript is structured: the Related Work section describes previous research on the segmentation of anatomical structures in X-ray images. The Proposed Approach section provides a detailed description of our method. In the Experiments and Results section, we perform a comprehensive evaluation of the proposed approach. The Discussion section analyses our approach and compares its performance with relevant studies. Finally, the Conclusions section summarizes the results and concludes concerning the proposed method.

## 2 Related work

The current state of research in the field of medical image segmentation, specifically for CXR, is progressing rapidly, particularly with the advent of deep learning techniques. The main focus of these studies includes improving segmentation accuracy, generalizability to new datasets, and algorithm efficiency. Among the prominent models used are convolutional neural networks (CNNs) and their variations, such as U-Net and its modifications, which are widely recognized for their ability to detect complex anatomical structures. In this section, we divide the analysis into two categories: segmentation and segmentation followed by classification.

### 2.1 Segmentation-only approaches

Thе work Nimalsiri et al. (2023) focuses on the creation of a large CXR-segmented dataset based on MIMIC-CXR and the use of the SA-UNet model for automatic segmentation of chest X-ray images. This approach incorporates spatial attention (SA) to improve segmentation, specifically aiding in the accurate extraction of lung regions. The main contribution of this study is the provision of a large-scale dataset with segmented masks, which can be utilized to train and refine deep-learning models for medical diagnostics. However, the focus of this study is on lung segmentation, and it does not fully address the segmentation of other anatomical structures such as the heart and clavicles, which are more challenging due to their complexity and lower contrast in X-ray images.

In contrast, our work proposes a novel approach that combines U-Net with contour extraction methods to improve the segmentation of low-contrast anatomical structures. Our method achieves superior segmentation results for the lungs, heart, and clavicles, outperforming previous studies in terms of accuracy, especially in challenging anatomical areas. The integration of contour analysis before applying deep learning enhances feature extraction, contributing to improved segmentation quality in complex areas, and further automating the process for medical diagnostics.

Further improvements to segmentation have been explored with various U-Net modifications. For example Wang et al. (2019) presented the Mask R-CNN network, which achieved impressive results for segmenting the lungs, heart, and clavicles compared to studies conducted before 2019. This method provided a Dice coefficient of 97.6% for the lungs, 94.9% for the heart, and 92% for the clavicles. Although the results for the heart and clavicles are slightly inferior to the lungs, Mask R-CNN shows the potential for high segmentation accuracy.

The next step in the evolution of the models was the introduction of improved versions of U-Net. In a study Ma et al. (2021), a modified U-Net model with multi-channel extended convolution and dense deep-layer aggregation was developed. This model achieved high performance for lung segmentation: Dice coefficient = 97.9% and Jaccard index = 95.61%, but needs further improvement to improve the segmentation accuracy of other organs. The study Lyu et al. (2021) presented the RU-Net model, which combines the U-Net and residual network architectures. Although the model demonstrated a satisfactory result of the Jaccard index = 85.57% for the heart, this value is not high enough and indicates the need for further improvements. In Liu et al. (2022), an improved U-Net architecture was presented that integrates the EfficientNet-b4 model as an encoder along with residual blocks and the LeakyReLU activation function. This model achieved high results for lung segmentation, including Accuracy = 98.55% and Dice coefficient = 97.92%. While these results are encouraging, segmentation of other organs such as the heart and clavicles may require further improvements. Similar approaches to modifying U-Net were continued by Chavan et al. (2022), where a new architecture, ResUNet++, was presented, combining U-Net segmentation networks with ResNet feature extraction. The model attained a Dice coefficient of 95.95% for lung segmentation, indicating a strong performance. However, additional refinements may be necessary to enhance its accuracy further.

In contrast to the improved versions of U-Net, other approaches, such as graph neural networks, also show promising results. For example, a study Gaggion et al. (2023a) presented a landmark-based HybridGNet model that uses graph representations to improve segmentation. The model demonstrated high results for the lungs, so the Dice coefficient = 98%, and lower results for the heart and clavicles, indicating the potential of graph-based methods to improve segmentation accuracy, but with high computational requirements. Also, in Gaggion et al. (2023b), a HybridGNet model was proposed that combines traditional convolutional methods with graph convolutional neural networks (GCNN). The model achieved a Dice coefficient of 97.4% for the lungs and 93.3% for the heart. This highlights the potential of graph-based methods in improving segmentation, but the results for the heart can still be improved. A new approach combining graph and dense segmentation methods was proposed by the authors (Bransby et al., 2023). The model achieved a Dice coefficient of 96.98% for lung segmentation and 94.51% for heart segmentation, indicating that further improvement is needed.

Significant progress in segmentation is also observed in dual coding-decoding systems. In Ullah et al. (2023), a framework is presented that uses the VGG19 model as an encoder and recurrent residual blocks to improve segmentation. Although the model demonstrated the following results for the lungs: Jaccard index = 95.5% and Dice coefficient = 97.6%, the results for the heart and clavicles were worse.

The paper Müller and Kramer (2021) presents the open source Python library MIScnn, which simplifies the process of creating medical segmentation pipelines. The main goal of the library is to provide an intuitive API for rapidly building medical segmentation pipelines, including data input and output, preprocessing, data augmentation, piecewise analysis, metric evaluation, and the use of advanced deep models for learning and prediction. MIScnn also allows for parameter tuning and flexibility in working with different CNN models, making it easy to quickly switch between architectures and add custom models. However, despite its flexibility and ease of use, this approach has limitations in the context of specific segmentation tasks, as most models are targeted at standard datasets and are not optimized for complex or specific cases, such as low-contrast medical images, where our method, which integrates contour detection with U-net, demonstrates significantly better accuracy and segmentation results for complex anatomical structures.

The recent advances in deep learning-based medical image segmentation are studied and analyzed by the authors in Rayed et al. (2024), with a focus on applications, algorithms and challenges in this field. This study provides a comprehensive analysis of recent applications, commonly used datasets, preprocessing methods, and deep learning algorithms, and analyses the state-of-the-art in deep learning medical image segmentation through experimental results.

In the current study Agarwal et al. (2024), a new approach for biomedical segmentation using a two-channel decoder and Attention-gated Swin transformers is proposed, which effectively extracts local and global image dependencies. The main advantage is the improvement of segmentation quality without increasing computational costs. However, despite the successful application of this model for the segmentation of liver and spleen tumors, its main disadvantage is the need for a large dataset for training, as well as potentially high computational costs due to the complexity of the transformer networks. Compared to our work, where we apply contour detection methods together with the U-Net model to improve the lung, heart, and clavicle segmentation accuracy in Chest X-ray images, our approach demonstrates high accuracy and does not require such a large amount of data or significant computational resources, making it more accessible and efficient for practical applications.

### 2.2 Segmentation followed by classification

The work Kumarasinghe et al. (2022) integrates a modified U-Net architecture for segmentation, followed by infectious disease classification based on the segmented regions. While this approach excels in classification accuracy, it only evaluates segmentation as a preliminary step to support classification tasks rather than improving the segmentation accuracy for each anatomical structure individually. In contrast, our study focuses solely on improving the segmentation itself, particularly for low-contrast anatomical structures such as the lungs, heart, and clavicles. By combining U-Net with contour extraction methods, we achieve superior segmentation accuracy without requiring post-processing steps typically used for classification.

Moreover, the Jafar et al. (2022) study, presenting the CardioNet model for multi-class segmentation, aims to more accurately segment the heart, lungs, and clavicles with fewer parameters. Although high results are obtained for the lungs (Dice coefficient = 98.61%), the heart and clavicles still present significant challenges (heart Dice coefficient = 94.76%, clavicles Dice coefficient = 92.74%). These results underscore the need for further improvements, especially in the segmentation of more anatomically complex regions.

Our approach, by focusing exclusively on enhancing segmentation accuracy for each anatomical structure, offers a more efficient and precise solution for applications in automated medical systems, bypassing the need for additional classification steps to achieve robust anatomical segmentation.

A recent study Hasan et al. (2024) provides valuable insights into segmentation and classification approaches, though they focus on histopathological images for breast cancer diagnosis.

This paper presents a hybrid model that integrates SegNet and U-Net architectures for the segmentation of histopathological images. The decoder is modified to incorporate ResNet, VGG, and DenseNet, which are used for classification tasks. The models were trained and evaluated using both private and public datasets. The proposed method achieved a high pixel-based segmentation F1-score of 0.902 for private datasets and 0.903 for public datasets, along with a classification F1-score of 0.833 for private datasets. This study demonstrates the effectiveness of hybrid models and the integration of various architectures to achieve high segmentation and classification performance in histopathological analysis. However, this approach is limited by its reliance on hybrid architectures and the need for further improvement in real-time applications, particularly in processing at higher magnifications.

The study Breesam et al. (2024) considers the importance of segmentation and classification of medical images using artificial intelligence methods, in particular convolutional neural networks (CNNs). Their role in detecting and identifying diseased areas and anatomical structures in medical images such as MRI, CT, and X-rays is described. In addition, it is noted that despite the difficulties associated with data privacy, the need for large annotated datasets, and the interpretability of models, artificial intelligence has the potential to improve diagnostic accuracy and clinical decision-making. In our research, we propose improvements in the segmentation of low-contrast structures such as clavicles using contour detection techniques integrated with the U-Net model to improve segmentation accuracy in medical chest images. We will focus on optimizing methods for segmenting complex anatomical regions and developing solutions for automating processes in medical diagnostics.

In summary, significant progress has been made in chest X-ray image segmentation, especially using deep learning techniques such as U-Net modifications and graph neural networks, but significant challenges remain in the accuracy of segmentation of anatomically complex areas such as the heart and clavicles. The studies reviewed show that the segmentation of structures such as the lungs has achieved high accuracy, but further advances are needed to improve the segmentation of more complex anatomical structures. Future research should focus on optimizing these models to both achieve high accuracy and reduce computational complexity, especially for clinical applications requiring real-time processing and automated analysis of complex areas.

## 3 Materials and methods

### 3.1 Segmentation algorithm for chest X-ray images

This research presents a new approach to chest X-ray image segmentation, which consists of enhancing X-ray images using edge detection algorithms before training the segmentation model. This preprocessing step is designed to improve the model’s ability to more accurately delineate the lungs, heart, and clavicles, which pose segmentation challenges due to their varying contrast and overlap in X-ray projections.

Edge detection is an important step in image processing, especially for extracting meaningful information from medical images, where the clarity of the edges can have a significant impact on diagnostic results. By applying edge detection algorithms suitable for capturing fine details in X-ray images, this method achieves several purposes:

1. Edge detection algorithms improve the visibility of boundaries between different anatomical structures by emphasizing intensity transitions, which are the hallmarks of edges.
2. Medical images, especially X-ray images, often contain a significant amount of noise. Edge detection helps to reduce the impact of this noise by focusing on significant transitions and ignoring insignificant variations in pixel values.
3. Thanks to clearer boundaries and reduced noise, the neural network training process becomes more efficient. The model can better learn to recognize and generalize from discernible features, instead of adjusting to noise or fuzzy edges.

After the edge detection process is applied to the X-ray images, the resulting images, which now display improved edges, serve as input to the convolutional neural network model.

First of all, a set of CXR is loaded along with the corresponding segmentation masks. Next, an edge detection algorithm is applied to the X-ray images. This step emphasizes the boundaries and contours of anatomical structures such as lungs, heart, and collarbones. Next, data augmentation is performed to increase the size of the dataset and improve the model’s robustness to variations in new, unseen data. The next step is data preprocessing to prepare the data for transmission to the network. After that, the model is trained using preprocessed images with enhanced edges as input and appropriate masks. The last step is to evaluate the model performance using metrics such as the Dice coefficient and the Jaccard index.

Below is a pseudo-code (see Algorithm 1) representation of the proposed segmentation algorithm, summarizing these processing steps in a structured format.

**ALGORITHM 1:**
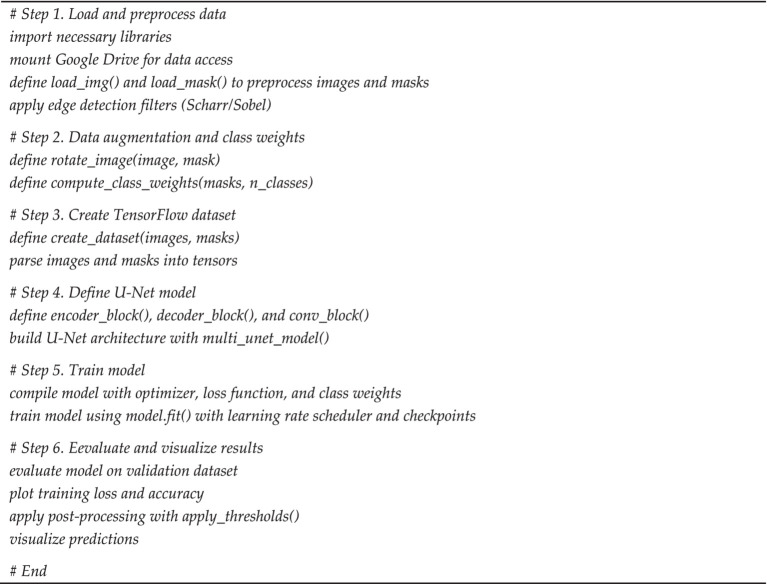
Pseudocode for image segmentation pipeline using U-Net.

### 3.2 Edge detection methods

In this paper, two edge detection methods are investigated: the Sobel filter and the Scharr filter. The choice of these two methods is motivated by their effectiveness and balance between computational efficiency and edge detection accuracy. Both methods effectively detect the edges of objects in images by highlighting important boundaries, which is particularly useful for segmentation, where clear boundary definition is essential to accurately delineate objects. Detected edges help segmentation algorithms better understand where one object ends and another begins.

The Sobel method is one of the most well-known edge detection algorithms that use two filters (matrices) to calculate brightness gradients for each pixel of an image. These gradients are used to detect areas where the intensity changes, which is a sign of the presence of edges. This method was chosen for its simplicity, computational efficiency, and ability to highlight significant edges, which makes it well-suited for medical image segmentation.

The Scharr filter was selected due to its increased sensitivity to small changes in intensity compared to Sobel. This method is known to provide sharper and more precise edge detection, especially in situations where fine boundaries are critical for accurate segmentation. While alternative methods such as Canny, Laplacian, or Rand exist, the focus on Sobel and Scharr in this study is based on their well-established performance in segmentation rather than on implementation complexity. These methods provide a balance between detection accuracy and computational efficiency, which is particularly relevant for medical applications requiring both precision and real-time processing capabilities, ensuring effective segmentation performance.

Imagine that we have an image where each pixel has a different brightness value. To detect changes in intensity, we use filters that calculate the difference in pixel intensities in the horizontal and vertical axes (X and Y axes). Gradients in both directions are calculated using the following formulas Equations 1,2:

1. Horizontal gradient $g_{x}$

(1) $$g_{x}\left(i,j\right)=\sum_{u=-1}^{1}\sum_{v=-1}^{1}I\left(i+u,j+v\right)\cdot g_{x}\left(u+1,v+1\right)\text{,}$$

2. Vertical gradient $g_{y}$

(2) $$g_{y}\left(i,j\right)=\sum_{u=-1}^{1}\sum_{v=-1}^{1}I\left(i+u,j+v\right)\cdot g_{y}\left(u+1,v+1\right)\text{,}$$

where $I\left(i,j\right)$ – is the intensity of the pixel at the point $\left(i,j\right)$ , and $g_{x}$ and $g_{y}$ are filter elements used to calculate gradients in the horizontal and vertical directions, respectively.

After calculating the gradients in both directions, you can determine the total gradient at each pixel as the combination of these two values Equation 3:


(3) $$g\left(i,j\right)=\sqrt{g_{x}{\left(i,j\right)}^{2}+g_{y}{\left(i,j\right)}^{2}}$$

This general gradient $g\left(i,j\right)$ shows the amount of intensity change at each pixel, allowing you to detect areas with a sharp transition between light and dark parts of the image, i.e., edges.

The Sharpe method is similar to the Sobel method, but it improves accuracy and maintains better rotational symmetry, allowing it to work better with more detailed image structures (Qian, 2024). To detect gradients, this method uses other filters that give more weight to the central pixels that are close to the target pixel. The corresponding filters for horizontal and vertical edge detection are as follows:

- Horizontal filter:

$$g_{x}=\left[\begin{array}{ccc} -3 & 0 & 3\\ -10 & 0 & 10\\ -3 & 0 & 3 \end{array}\right]$$

- Vertical filter:

$$g_{y}=\left[\begin{array}{ccc} -3 & -10 & -3\\ \;0 & \;0 & \;0\\ \;3 & \;10 & \;3 \end{array}\right]$$

Similarly to the Sobel method, the horizontal and vertical gradients are calculated for each pixel using these filters, and then the total gradient for each pixel is calculated using the Equation 6.

Both of these methods help us to find edges in images, which is important for accurately isolating anatomical structures such as lungs, heart, and collarbones in chest X-ray images. Clear edge detection can significantly improve the results of subsequent segmentation, as the model better recognizes and separates these structures, even if they have similar pixel intensities or overlap with other structures.

### 3.3 U-Net model architecture

The U-Net model is widely used due to its efficiency and effectiveness in solving various image segmentation problems, especially in the field of medical imaging (Ronneberger et al., 2015). The U-Net architecture is characterized by a symmetrical “U-shaped” design, which consists of two main parts: compression (encoder) and expansion (decoder).

In the context of chest X-ray segmentation, U-Net is particularly well suited for several reasons:

- The model’s ability to work at different scales makes it effective in recognizing the fine details needed to accurately delineate anatomical structures such as lungs, heart, and collarbones.
- Medical images can vary greatly in terms of structure visibility due to different patient positions, pathologies, or imaging conditions. U-Net’s robust learning and generalization capabilities make it effective in handling this variability.
- For this work, the integration of edge enhancement techniques before image input into the U-Net model can further improve its ability to accurately detect and segment relevant medical structures. By using edge detection to enhance the boundaries, the U-Net model is able to capture fine details and improve segmentation accuracy.

The model uses a sigmoid activation function on the output layer. In this case, classes can overlap (i.e., one-pixel region can simultaneously belong to several classes), so using a sigmoid activation function for each channel of the output layer may be a better option than the softmax function.

The formula of the sigmoid function (Xu et al., 2021; Kovtun et al., 2023):


$$\sigma \left(x\right)=\frac{1}{1+e^{-x}}$$

where x is the input signal to the neuron.

The sigmoid activation function processes each output channel independently. Each channel of the output layer represents the probability that a pixel belongs to a certain class. For example, if the output layer has three channels for three different classes (lungs, heart, collarbone), each channel gives the probability of belonging to each of these classes.

In the semantic segmentation problem, when there are classes such as lungs, heart, and collarbones in medical images, the dataset is considered unbalanced. Despite the fact that each image contains all three classes, the lungs in X-ray images occupy a much larger area compared to the heart and collarbones. This means that the number of pixels representing the lungs is much higher than the number of pixels for the heart and collarbones. Thus, it is important to calculate class weights to make the model more sensitive to classes that are represented by less data.

The calculation of class weights is based on the idea of inverse weighting of class frequencies in a dataset (Sugino et al., 2021):

- The class frequency is defined as the ratio of the number of occurrences of a particular class to the total number of occurrences of all classes.
- The class weight is inversely proportional to the class frequency. This means that less-represented classes are assigned higher weights, which helps compensate for their lower frequency and ensures that the model prioritizes them during processing.

This ensures that the model does not favor any class at the expense of others, while giving more attention to the less frequently occurring classes.

To calculate the loss value, Categorical Focal Cross entropy is used, which is based on Focal Loss (Hadinata et al., 2023). This function was developed to improve the efficiency of model training on data with strong class imbalance, especially in segmentation problems. The main idea is to modify the usual cross-entropy in such a way as to reduce the influence of easy-to-classify examples on the overall loss function and focus the model’s attention on more difficult examples. Mathematically, this is implemented using the following mechanism:


$$\mathrm{Focal}\;\mathrm{Loss}=-\alpha_{t}{\left(1-p_{t}\right)}^{\gamma }\log\left(p_{t}\right)$$

where $p_{t}$ is the probability of the correct class for the current pixel; $\alpha_{t}$ is the class weight, which helps to compensate for class imbalance (less represented classes receive higher weight); γ is the focusing parameter that controls how strongly the model focuses on difficult examples by reducing the attention to easy ones.

This mechanism allows the model to concentrate on examples that are difficult to classify or have a higher error rate, improving overall model performance in the presence of class imbalance. The use of class weights ensures that the model pays more attention to underrepresented classes, thus providing more balanced training across all stages.

As for the learning rate of the model, the value of the learning rate is regulated by a polynomial decay function. The formula for calculating the learning rate (Boulaaras, 2020) is as follows:


$$\begin{array}{l} \mathrm{learnin}g_{\mathrm{rate}}=\left(\mathrm{initia}l_{\mathrm{learnin}g_{\mathrm{rate}}}-end_{\mathrm{learnin}g_{\mathrm{rate}}}\right)\\ {\left(1-\frac{\mathrm{step}}{\mathrm{deca}y_{\mathrm{steps}}}\right)}^{\mathrm{power}}+end_{\mathrm{learnin}g_{\mathrm{rate}}} \end{array}$$

where $\mathrm{initia}l_{\mathrm{learnin}g_{\mathrm{rate}}}$ is the initial learning rate; $end_{\mathrm{learnin}g_{\mathrm{rate}}}$ is the final learning rate; step is the current training step; $\mathrm{deca}y_{\mathrm{steps}}$ is the number of steps (epochs) during which the learning rate will decrease; power is the polynomial degree. The use of this function provides a smoother and less aggressive decrease in the learning rate, which can contribute to better model convergence in the final stages of training.

The methodology for medical image segmentation in this study involves enhancing X-ray images using edge detection techniques before training a CNN for segmentation. The model is trained with preprocessed images that have enhanced edges, along with corresponding segmentation masks, to ensure accurate delineation of anatomical structures.

The training process starts with loading the chest X-ray images and their associated segmentation masks, which highlight key structures like the lungs, heart, and clavicles. These masks are essential for guiding the model’s learning process.

Edge detection algorithms, such as Sobel and Scharr filters, are applied to the X-ray images to enhance boundary definition between anatomical structures. These methods were chosen based on their effectiveness in highlighting intensity transitions, which aids in accurate segmentation by providing clearer structural differentiation.

Data augmentation follows, increasing the dataset’s variability by applying transformations such as rotation, reflection, and shifting. This step enhances the model’s robustness, allowing it to generalize better to unseen data.

Next, the images are preprocessed by normalizing pixel values, resizing them to a uniform size, and preparing them for input into the CNN. During training, the model learns to associate pixel patterns from the enhanced X-rays with corresponding mask patterns representing anatomical structures.

The loss function used during training is a combination of categorical cross-entropy and Focal Loss, which addresses class imbalance by focusing the model’s attention on harder-to-classify examples. An optimizer adjusts the model’s weights through backpropagation, improving the model’s segmentation performance.

Finally, the model’s performance is evaluated using metrics like the Dice coefficient and the Jaccard index, which provide quantitative measures of the model’s ability to segment anatomical structures accurately.

**Figure 1:**
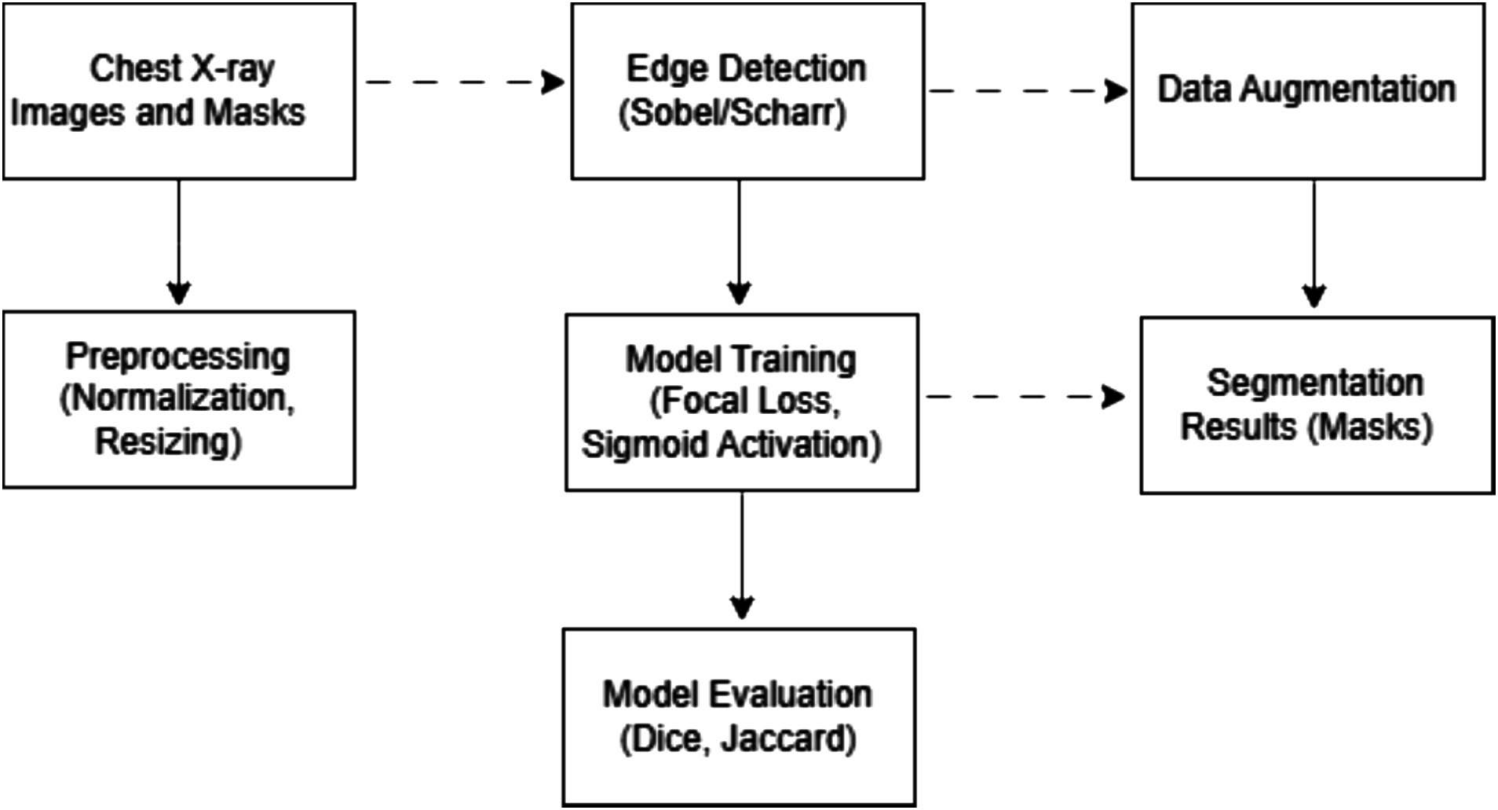
Architecture for model training for chest X-ray image segmentation.

Figure 1 illustrates the training process architecture, detailing the sequence of steps from data loading and edge detection to training and evaluation.

### 3.4 Evaluation metrics

To support our methodological choices, we formalize the segmentation problem as a mathematical optimization task. To improve the accuracy of semantic segmentation of medical X-ray images, we set up a mathematical optimization problem. Given a set of medical images $I=\left\{I_{1},\;I_{2},\ldots ,\;I_{n}\;\right\}$ , where each image $I_{i}$ contains several target regions corresponding to three classes (lungs, heart, collarbone), the problem is to find a segmentation function *S* that maps each image to a label map, maximizing the segmentation accuracy. The segmentation function *S* should be optimized to minimize the difference between the predicted segmentation and the ground truth segmentation. The truth for each image $I_{i}$ is given in the form of $G_{i}$ , which is a label map indicating the exact class boundaries.

The Dice coefficient and Jaccard index are popular metrics for evaluating the quality of image segmentation, particularly in multi-class segmentation problems.

The Dice coefficient is calculated as follows (Shamir et al., 2019) Equation 4:


(4) $$D\left(S\left(I_{i}\right),G_{i}\right)=\frac{2\times |S\left(I_{i}\right)\cap G_{i}|}{\left|S\left(I_{i}\right)+G_{i}\right|}$$

The D estimates the overlap between the predicted segmentation *S(* $I_{i}$ *)* and the truth $G_{i}$ . The aim is to maximize the D for all images in the set 𝐼.

The Jaccard index measures the proportion of overlap between the predicted and true classes in relation to their combination (Costa, 2021) Equation 5:


(5) $$J\left(S\left(I_{i}\right),G_{i}\right)=\frac{\left|S\left(I_{i}\right)\cap G_{i}\right|}{\left|S\left(I_{i}\right)\cup G_{i}\right|}$$

where *J* is the Jaccard index, *S(* $I_{i}$ *)* is the predicted segmentation, and $G_{i}$ is the ground truth segmentation. The aim is to maximize the J for all images in the set 𝐼.

Thus, the main mathematical aim is to solve the following optimisation problem Equation 6:


(6) $$\max_{S}\left(\frac{1}{n}\sum_{i=1}^{n}D\left(S\left(I_{i}\right),G_{i}\right)+\frac{1}{n}\sum_{i=1}^{n}J\left(S\left(I_{i}\right),G_{i}\right)\right)$$

where *S* is a segmentation function that takes an input image and outputs a segmentation map; *n* is the number of images in the dataset; $D\left(S\left(I_{i}\right),G_{i}\right)$ is the Dice Coefficient that estimates the similarity between the predicted segmentation $S\left(I_{i}\right)$ and the ground truth segmentation $G_{i}$ for the *i*-th image; $J\left(S\left(I_{i}\right),G_{i}\right)$ is the Jaccard index for the *i*-th image. By explicitly formulating the problem in mathematical terms, we emphasize the theoretical foundation of our approach. This formulation emphasizes the aim of improving the model’s segmentation accuracy by maximizing the values of both the Dice coefficient and the Jaccard index.

## 4 Experiments and results

### 4.1 Experimental setup and dataset details

The research was conducted using the Google Colab environment, which provides access to high-performance computing resources without the need to use your hardware. This ensured efficient performance of computations related to deep learning, as well as training models with high-performance requirements. To accelerate the computations, an NVIDIA T4 GPU with 15 GB of video memory was used, which significantly increased the speed of neural network training and the efficiency of task execution.

Python (version 3.x) was chosen as the main programming language for the experiments, as it supports a wide range of libraries for scientific computing and machine learning. To build and train the U-Net model, we used the TensorFlow library, which provides effective tools for working with neural networks, including GPU-optimized operations and flexible data management. The OpenCV library was used to process medical images, allowing for filtering, transformations, and contour detection.

Libraries were used for data processing: NumPy for numerical calculations and manipulations with tensors, SciPy for complex mathematical operations and optimization, Pandas for analysis and processing of structured data, and Matplotlib for visualization of image preprocessing and segmentation results. This configuration ensured efficient workflow organization for medical image segmentation, combining deep learning and computer vision methods with hardware acceleration.

The study used the JSRT dataset (JSRT Database, 2024), which was created by the Japan Society of Radiological Technology and is one of the classic datasets for analyzing CXR. It is widely used in medical imaging research, especially for detection and segmentation problems. The dataset includes 246 high-resolution CXRs. The images have a size of 2048×2048 pixels, which provides high detail for analysis.

The SCR (Segmentation in Chest Radiographs) dataset (van Ginneken, 2024) is a supplement to the JSRT dataset specifically designed for segmentation problems. It is designed to provide segmentation masks that can be used for training and testing deep learning models. For each image from JSRT, SCR provides corresponding masks that show the location and contours of key anatomical structures such as lungs, heart, and collarbones. The masks are provided in a 1024×1024 TIF format.

Figure 1 shows a flowchart illustrating the sequence of processing the X-ray images and masks. The data processing process for medical image segmentation in this study involves a complex sequence of steps, each of which is critical to preparing the data and ensuring optimal performance of the segmentation model. Below is a detailed description of each step:

1. Data download.

- The initial stage of downloading high-resolution CXR and associated masks.

2. Combine mask.

- Separate masks for different anatomical structures (left and right lungs, clavicles) are combined into one mask for each structure to simplify the segmentation process. An additional background channel is created for high-quality segmentation.

3. Edge Detection.

- Edge detection algorithms such as Sobel and Scharr filters are applied to enhance the visibility of anatomical boundaries, as these methods effectively highlight transitions in intensity, which is essential for delineating structures in medical imaging.

4. Augmentation.

- Augmentation methods are used to increase the model’s robustness to variations in new images. In this case, the image and the mask are rotated by a certain angle in the range from −5 ° to 5 °, except for 0 °. After data augmentation, the size of the dataset contained 492 images.

5. Preprocessing.

- All images and masks are resized to 512 × 512. This is important to ensure that all images are processed equally by the model. Image pixel values $I\left(i,j\right)$ are normalized to the range from 0 to 1: $0\leq I\left(i,j\right)\leq 1$ .

6. Separation into training and test samples.

- The dataset is divided into training and test sets in the ratio of 80:20.

7. Model training.

- Using preprocessed and supplemented data, the U-Net neural network model is trained. During the training, the model learns to associate certain pixel patterns of X-ray images with corresponding mask patterns indicating different anatomical structures.

8. Segmentation.

- After training, the model is used to segment new X-ray images. It applies the learned patterns to predict anatomical structures in the images, creating segmentation masks as an output.

Several experiments were conducted to analyze the impact of edge detection methods on the performance of the medical image segmentation model. First, the U-Net model was trained on the original images without applying any edge enhancement techniques. This allowed us to establish a baseline for comparing the model’s performance.

Next, we applied one of the edge detection methods, the Sobel algorithm, to the images before training the model. Images with enhanced edges were used to train the model, and then key performance metrics such as the Dice and Jaccard coefficients were calculated to evaluate the segmentation accuracy. After that, the process was repeated with another edge detection method, namely the Scharr algorithm, applying it to the input images. After training the model on the resulting images, the metrics are again measured to compare the performance of the different approaches.

### 4.2 Ablation experiments: impact of edge detection on segmentation

**Figure 2:**
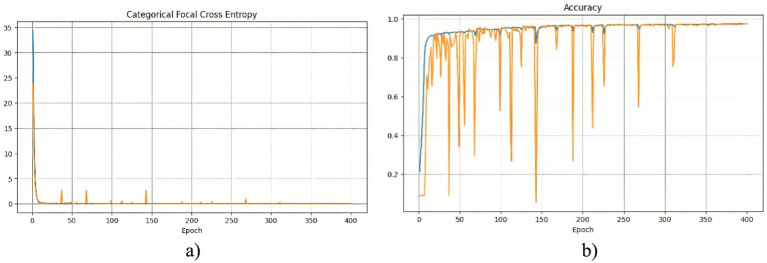
Training results of the model without using edge detection methods: **(a)** training and validation loss; **(b)** training and validation accuracy.

The segmentation model was trained for 400 epochs using the Polynomial Decay algorithm to reduce the learning rate. This method allowed us to gradually reduce the learning rate with each epoch, which contributed to better convergence to the minimum of the loss function during model training. The model training process lasted about 2 h. The effectiveness of the training process can be analyzed based on the changes in the loss function and accuracy, which are presented in Figure 2.

**Figure 3:**
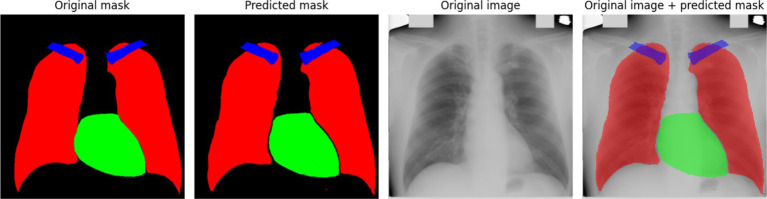
An example of model prediction without using edge detection methods.

At the beginning of training, a rapid decrease in the loss function is observed (Figure 2a), indicating that the model quickly learns meaningful features from the data. After this initial drop, the loss stabilizes at a low level, suggesting that the model reaches an optimal state. However, periodic fluctuations are noticeable, which may be caused by variations in the complexity of different training batches. These peaks indicate moments when the model encounters more challenging examples, possibly leading to temporary increases in error. The accuracy curve (Figure 2b) shows a generally increasing trend, reaching high values. However, sharp declines in accuracy are observed throughout training. These fluctuations could be caused by batch variability or overfitting to certain parts of the training set. Despite these occasional drops, the overall accuracy stabilizes at a high level, indicating that the model successfully generalizes across most of the dataset. To further assess the quality of the model’s predictions, Figure 3 presents a visual comparison between the ground truth segmentation and the predicted masks.

The predicted segmentation mask closely resembles the original mask; however, the contours of the anatomical structures appear less distinct. This observation aligns with the quantitative results, confirming that the absence of edge detection methods reduces segmentation precision. The blurry contours in the predicted mask indicate that the model has difficulty defining sharp anatomical boundaries, particularly for smaller structures like the clavicles. These findings suggest that additional preprocessing techniques, such as edge detection, may be beneficial in improving segmentation accuracy.

**Figure 4:**
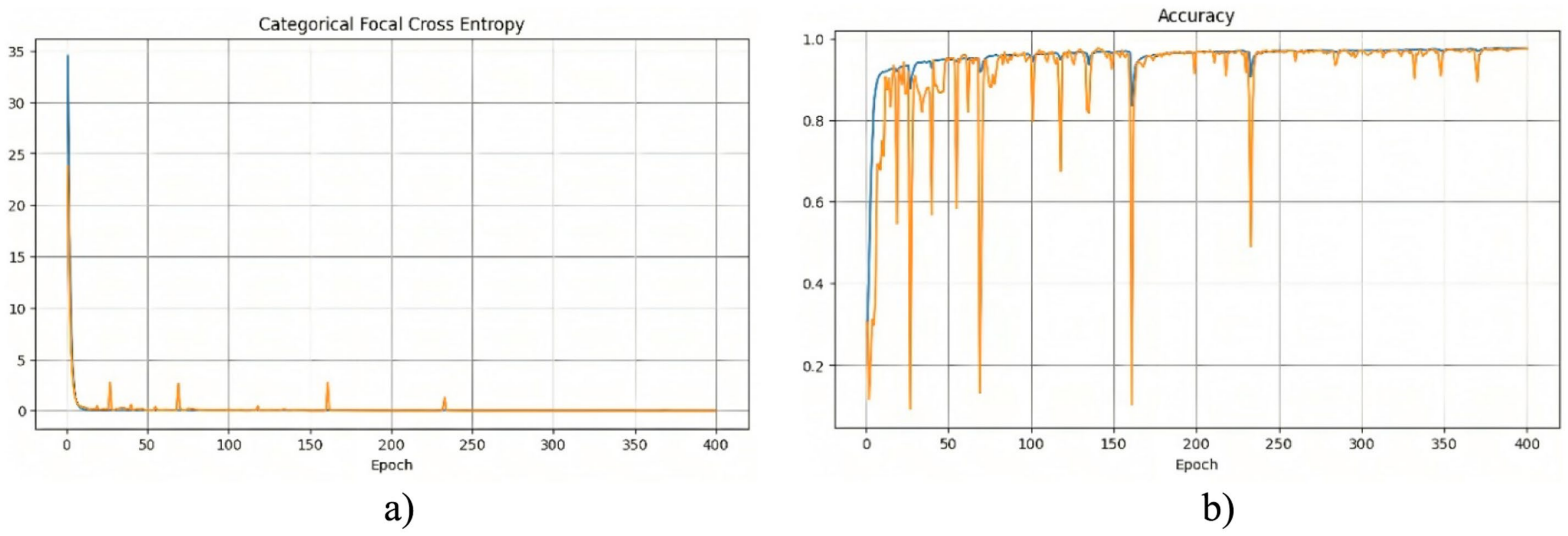
Training results of the model using the Sobel method: **(a)** training and validation loss; **(b)** training and validation accuracy.

The segmentation model was similarly trained for 400 epochs using the Polynomial Decay algorithm to reduce the learning rate. Figure 4 consists of two subfigures: (a) training and validation loss, and (b) training and validation accuracy, illustrating the changes in the loss function and accuracy over the training process, demonstrating the model’s learning behavior and performance stabilization.

A noticeable sharp drop in losses at the beginning of the training indicates that the model quickly adapts to the training data. Following this rapid decline, the losses stabilize, with occasional peaks suggesting instances of increased error, possibly due to challenging cases in the dataset. At the start of training, the accuracy graph exhibits large fluctuations, indicating that the model is highly sensitive to variations in the data. After approximately 80 epochs, the accuracy stabilizes at a high level, with occasional minor drops, demonstrating the model’s ability to generalize effectively.

**Figure 5:**
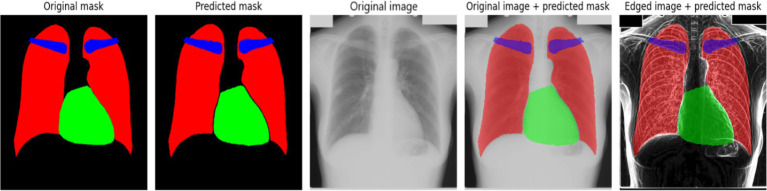
An example of model prediction using the Sobel method.

Figure 5 provides a comparative visualization of the model’s segmentation results, showcasing both the predicted masks and their overlay with the original X-ray images. The predicted masks align closely with the original masks, confirming the high quality of the model’s segmentation. Furthermore, when overlaid on the X-ray images, the segmented structures correctly follow anatomical boundaries, demonstrating the model’s effectiveness in distinguishing different regions. The use of the Sobel method contributes to sharper contours, which is particularly evident when compared to segmentation results without edge enhancement. This highlights the advantage of integrating edge detection techniques into deep learning-based medical image segmentation.

**Figure 6:**
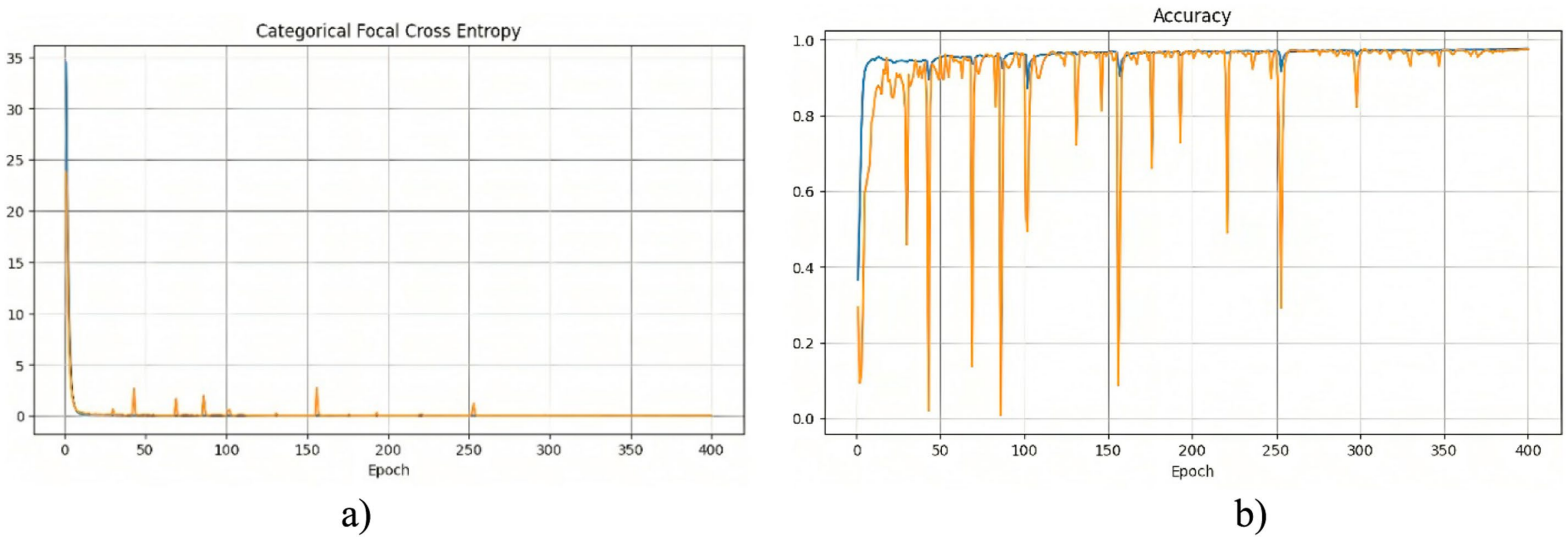
Training results of the model using the Scharr method: **(a)** training and validation loss; **(b)** training and validation accuracy.

The segmentation model was similarly trained for 400 epochs, using the Polynomial Decay algorithm to reduce the learning rate. Figure 6 presents the variations in the loss function and accuracy throughout the training process.

**Figure 7:**
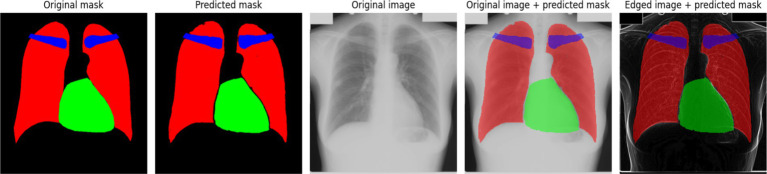
An example of model prediction using the Scharr method.

The results depicted in Figure 6a indicate that the training and validation loss initially decrease sharply, followed by a stabilization phase with occasional spikes. These spikes may correspond to instances where the model encounters new or more challenging samples in the dataset. This behavior suggests that the model adapts dynamically to the variability in training data, improving its generalization capabilities over time (Figure 6b) illustrates the accuracy progression during training. At the early training stages, significant instability is observed, which could be attributed to sensitivity to weight initialization or the complexity of the dataset. However, after the initial fluctuations, the accuracy stabilizes at a high level, indicating that the model effectively learns the segmentation task. Figure 7 presents an example of model predictions using the Scharr method. The predicted masks demonstrate a high level of agreement with the original ground-truth masks. The lungs and heart segmentation exhibit some minor contour irregularities, yet the overall result is visually superior to segmentation performed without edge detection. The enhanced delineation of structures suggests that integrating edge detection helps in refining the model’s performance by emphasizing boundaries and reducing noise, leading to better anatomical segmentation.

Table 1 provides a comparative analysis of the segmentation performance across different anatomical structures using three different approaches: the baseline model (without edge detection), and the Sobel and Scharr edge detection methods. The performance metrics accuracy, Dice coefficient, and Jaccard index are presented for each anatomical class: lungs, heart, and clavicles. This allows for a detailed evaluation of how each edge detection technique affects the segmentation results, particularly for smaller structures like the clavicles.

**Table 1:** Comparison of segmentation results without edge detection methods and with the use of Sobel and Scharr methods.

Class	Edge detection method	Accuracy	Dice coefficient	Jaccard index
Lungs	No edge detection	98.52	97.46	95.07
	Sobel	99.26	98.88	97.54
	Scharr	98.81	98.19	96.25
Heart	No edge detection	99.08	94.78	90.62
	Sobel	99.47	96.96	94.14
	Scharr	99.26	96.63	92.54
	No edge detection	99.52	88.06	78.84
Clavicles	Sobel	99.79	94.43	89.57
	Scharr	99.66	91.72	85.04

As shown in Table 1, the Sobel edge detection method consistently outperforms both the baseline model and the Scharr method regarding accuracy, Dice coefficient, and Jaccard index across all anatomical structures. Notably, the Sobel method significantly improves segmentation of the lungs and heart, yielding high values for both Dice and Jaccard metrics. For clavicles, while the Sobel method also shows an improvement, its segmentation remains less precise compared to the larger structures, likely due to the clavicles’ smaller size and less distinct edges.

The Scharr method, on the other hand, demonstrates a noticeable improvement over the baseline model, particularly for the clavicles, although its performance still falls slightly behind the Sobel method. Despite this, the accuracy for the clavicles remains the highest among all three methods, suggesting that the Scharr method is effective for structures with more explicit boundaries but may require further refinement for smaller or more complex anatomical structures. Overall, these results highlight the benefits of incorporating edge detection techniques to refine segmentation boundaries, with Sobel providing the most consistent improvements across the board.

Overall, the results indicate that the Scharr method effectively improves segmentation accuracy while maintaining computational efficiency. However, further fine-tuning and additional post-processing techniques may be beneficial for refining the segmentation of complex structures such as the clavicles.

The results of experiments with edge detection using the Sobel and Scharr methods showed an improvement in segmentation accuracy, in particular, in detecting organ boundaries. However, despite the improvement, there are still artifacts that affect the segmentation accuracy. In future experiments, we will focus on applying additional methods to reduce these artifacts and improve segmentation results.

### 4.3 Comparative assessment of edge detection methods and validation strategies in segmentation

To further assess the impact of edge detection methods on segmentation accuracy, additional experiments were conducted using the COVID-19 Radiography Database (Agarwal et al., 2024) and the Shenzhen Hospital X-ray Set (Raddar, n.d.). The objective was to determine whether edge detection methods, specifically Sobel and Scharr, introduce artifacts that negatively affect segmentation, particularly in small datasets.

To evaluate the potential artifacts caused by edge detection, preliminary contour detection was performed using the Sobel and Scharr algorithms. The original images were compared with their processed versions, and changes in frequency components were analyzed. The results indicated that in some cases, edge detection led to noise amplification and over-emphasis on small objects, including minor artifacts or contours not belonging to primary anatomical structures.

Some artifacts, particularly in regions with small or poorly defined structures (e.g., clavicles), were mitigated by additional filtering techniques such as CLAHE (Contrast Limited Adaptive Histogram Equalization) (Yussof et al., 2022). Furthermore, adaptive thresholding (Roy et al., 2014) was applied post edge detection to minimize the adverse effects of artifacts. This approach reduced noise by enhancing only the most significant contours while preventing over-segmentation of minor elements, which can introduce segmentation errors. The CLAHE method was employed to improve image contrast, effectively compensating for uneven illumination and enhancing local details, which is particularly beneficial for medical imaging. Adaptive thresholding helped suppress noise and artifacts resulting from edge detection, leading to more precise delineation of anatomical structures.

Experiments demonstrated that adaptive thresholding significantly reduced the number of artifacts and improved segmentation accuracy. A comparative analysis of segmentation performance with and without thresholding is presented in Table 2.

**Table 2:** Segmentation results for different edge detection methods with adaptive thresholding on two datasets.

Edge detection method	Class	Accuracy (%) (COVID-19 radiography)	Dice coefficient (COVID-19 radiography)	Jaccard index (%) (COVID-19 radiography)	Accuracy (%) (Shenzhen hospital)	Dice coefficient (Shenzhen hospital)	Jaccard index (%) (Shenzhen hospital)
No edge detection	Lungs	98.52	97.46	95.07	97.94	96.48	93.56
Sobel + adaptive thresholding	Lungs	99.14	98.21	96.89	98.46	97.11	94.63
Scharr + adaptive thresholding	Lungs	99.32	98.55	97.12	98.64	97.35	94.94

These findings suggest that adaptive thresholding effectively mitigates the negative impact of artifacts while preserving the benefits of edge detection. The results obtained from the Shenzhen Hospital X-ray Set further validate the robustness of this approach, demonstrating improved segmentation accuracy across different datasets.

Additional numerical experiments confirmed that while edge detection enhances contour visibility, it may also introduce noise, particularly in small structures. However, adaptive thresholding mitigates these effects by refining the most critical contours and reducing over-segmentation of minor artifacts. This approach ensures a balance between contour enhancement and artifact suppression, ultimately improving segmentation performance in medical imaging applications.

For evaluating the stability and reliability of the results, the Shenzhen Hospital X-ray Set was chosen. This dataset is important for chest radiograph (CXR) analysis and was selected due to its diversity and ability to provide generalized results when applied to various types of medical X-ray images. The Shenzhen Hospital X-ray Set allows for assessing the model’s ability to adapt and its robustness when applied to images of different origins and characteristics.

To evaluate the stability of the results, a 10-fold cross-validation was performed on the Shenzhen Hospital X-ray Set. This process allowed testing whether the model’s accuracy was maintained across different data subsets. Each of the 10 splits provided independent model testing, enabling the calculation of average accuracy, Dice coefficient, and Jaccard index across all splits. The results of the 10-fold cross-validation for both Sobel and Scharr methods are shown in Table 3.

**Table 3:** Results of 10-fold cross-validation for the Sobel and Scharr methods (Shenzhen hospital X-ray set).

Class	Accuracy (%) (Sobel)	Accuracy (%) (Scharr)	Dice coefficient (Sobel)	Dice coefficient (Scharr)	Jaccard index (%) (Sobel)	Jaccard index (%) (Scharr)
Lungs	99.12	98.92	98.62	97.75	97.04	95.80
Heart	99.30	99.12	97.12	96.01	94.54	93.75
Clavicles	99.78	99.63	94.60	93.42	89.84	88.57

Stratification was also applied when splitting the data into training and test sets for both methods. This ensured that the class proportions were preserved in each subset, which is crucial for ensuring a balanced representation of all classes during testing. Stratification ensures that each class is adequately represented, reducing the potential for biased results due to uneven class distribution in the sample. The results of applying stratification for both Sobel and Scharr methods are presented in Table 4.

**Table 4:** Results with stratification for the Sobel and Scharr methods (Shenzhen hospital X-ray set).

Class	Accuracy (%) (Sobel)	Accuracy (%) (Scharr)	Dice coefficient (Sobel)	Dice coefficient (Scharr)	Jaccard index (%) (Sobel)	Jaccard index (%) (Scharr)
Lungs	99.10	98.82	98.54	97.60	96.92	95.61
Heart	99.36	99.20	96.80	95.89	94.05	93.55
Clavicles	99.75	99.55	93.87	92.94	88.99	87.87

The results showed that the application of these strategies improves the stability of the results and allows for a better assessment of the overall model performance for both methods. The use of stratification helped maintain class balance in the subsets, which is particularly important for medical images where certain classes may be underrepresented. This ensures more even training of the model and enhances the accuracy and stability of the results.

## 5 Discussion

### 5.1 Segmentation performance on JSRT + SCR dataset

Table 5 presents the segmentation performance of the proposed method and baseline approaches on the JSRT + SCR dataset, which was used for both training and evaluation. This provides a controlled comparison within the same experimental setup, allowing us to quantify the direct improvements introduced by edge detection techniques.

**Table 5:** Summary of X-ray image segmentation results using different edge detection methods.

Method	Lungs			Heart			Clavicles		
	Acc	Dice	Jaccard	Acc	Dice	Jaccard	Acc	Dice	Jaccard
U-net	98.52	97.46	95.07	99.08	94.78	90.62	99.52	88.06	78.84
U-net + Sobel filter	**99.26**	**98.88**	**97.54**	**99.47**	**96.96**	**94.14**	**99.79**	**94.43**	**89.57**
U-net + Scharr filter	98.81	98.19	96.25	99.26	96.63	92.54	99.66	91.72	85.04

The bold values in the table represent the highest accuracy (Acc), Dice coefficient, and Jaccard index for each anatomical region (Lungs, Heart, and Clavicles) among the tested methods.

The results in Table 5 demonstrate that the integration of the Sobel filter with U-Net consistently outperforms the baseline U-Net model across all anatomical structures. While these results confirm the effectiveness of edge detection, an independent evaluation is necessary to validate the generalization capability of the proposed approach.

### 5.2 Generalization to an independent dataset

To address this, additional testing was conducted on an external dataset (Shenzhen Hospital X-ray Set), as shown in Table 6.

**Table 6:** Comparison of segmentation methods on the independent Shenzhen hospital X-ray set dataset.

Method	Additional algorithms	Dice (Lungs)	Dice (Heart)	Dice (Clavicles)
Proposed (U-Net + Sobel)	Sobel	**98.62**	**97.12**	**94.60**
Proposed (U-Net + Scharr)	Scharr	97.75	96.01	93.42
Baseline U-Net	None	96.48	94.76	88.06
U-Net++ (own implementation)	Attention	97.89	96.03	92.80
RU-Net (own implementation)	Residual blocks	97.50	95.60	91.50

The bold values represent the highest Dice coefficient (Dice) for each anatomical region (Lungs, Heart, Clavicles) among the tested methods.

To verify the generality of the proposed method, all models were tested on an independent dataset. The results confirm that the U-Net + Sobel approach maintains the highest segmentation accuracy for all evaluated anatomical structures. This supports the claim that edge detection improves boundary accuracy, especially in complex anatomical regions such as clavicles. The use of an external dataset ensures that the observed improvements are not dataset specific, but rather reflect the robustness of the proposed approach.

### 5.3 Research questions analysis

Next, we will analyze how the results obtained in the study correspond to the research questions posed.

RQ1: The results obtained with the Sobel and Scharr filters confirm the importance of integrating these methods into the U-Net model. Integrating the Sobel filter resulted in significant improvements in all metrics for lung, heart, and clavicle segmentation compared to the baseline U-Net model.

RQ2: Comparison of the proposed approach with other state-of-the-art methods showed that the U-net + Sobel filter method outperforms all categories for all anatomical structures, including lung, heart, and clavicle segmentation.

RQ3: The results of the clavicle segmentation indicate that although the proposed method improves accuracy compared to the baseline U-Net model, further improvement is needed for this region. This can be explained by the variability of the anatomical structures in this area and the insufficient contrast between cellular structures, which requires additional methods to more accurately account for these features.

### 5.4 Limitations

In this study, the proposed method was compared with both the baseline U-Net model and its modifications (U-Net++, RU-Net), which are among the most widely used architectures for medical image segmentation. The comparative analysis demonstrated that the integration of edge detection techniques improves segmentation accuracy, particularly in boundary delineation. While further comparisons with attention-based and Transformer-based models could be considered in future research, the current results already provide strong evidence of the effectiveness of the proposed approach.

To validate the significance of the improvements, a paired t-test was conducted between the proposed U-Net + Sobel method and the baseline U-Net model. The results confirmed that the improvements in Dice coefficient were statistically significant (*p* < 0.05), demonstrating that the observed enhancements are not due to random variations but rather to the integration of edge detection techniques.

### 5.5 Future research directions

Additional preprocessing steps, such as filtering using contour detectors, can significantly affect the overall execution time of the segmentation algorithm. In our study, two filters were used to detect contours before the main segmentation stage: the Sobel filter and the Scharr filter. Although these steps increase the preprocessing time, their role in improving segmentation accuracy, in particular, in improving the visibility of anatomical structure boundaries, is significant.

To evaluate the impact of additional preprocessing steps on the runtime, we conducted a series of tests comparing the processing time with and without the use of the contouring step. The results showed that the processing time with the Sobel filter for contouring increased by 14.8% compared to the baseline method, and the processing time with the Scharr filter increased by 17.6%. For example, for one of the test images, the segmentation time without preprocessing was 2.5 s, with the Sobel filter – 2.87 s, and with the Scharr filter – 2.94 s.

The overall execution time of the method with preprocessing steps remained at an acceptable level for practical applications. However, future work should focus on optimizing preprocessing time while maintaining segmentation quality. One potential approach could involve using more efficient implementations of edge detection algorithms or integrating lightweight attention mechanisms to enhance feature extraction without significant computational overhead.

## 6 Conclusion

In this study, an integration strategy combining edge detection techniques with a deep learning model was implemented to enhance medical image segmentation. The results confirmed that incorporating Sobel and Scharr filters into the U-Net architecture significantly improves segmentation accuracy, particularly in delineating anatomical boundaries in chest X-ray images. The Sobel method proved to be the most effective in reducing artifacts, while the Scharr filter showed potential for detecting finer structures. The proposed approach demonstrated strong generalizability, validated on an independent dataset (Shenzhen Hospital X-ray Set).

Future research should focus on exploring the applicability of this approach to other modalities, such as MRI and CT, ensuring its robustness across diverse clinical settings. Additionally, further analysis of the impact of imaging artifacts and noise is necessary to refine preprocessing techniques. Furthermore, the reliance on predefined edge detection filters may limit adaptability to varying image qualities, suggesting the need for trainable edge detection mechanisms. Clinical validation is also essential to confirm the practical applicability of this method. Future work should also concentrate on enhancing model explainability through post-hoc visualization techniques to improve interpretability for medical professionals (Chumachenko and Yakovlev, 2025).

Future studies should investigate hybrid architectures, such as Transformer-based models, to further enhance performance. The proposed approach offers a promising direction for medical imaging applications, with potential benefits for disease diagnosis, treatment planning, and patient monitoring.

## Data Availability

The original contributions presented in the study are included in the article/supplementary material, further inquiries can be directed to the corresponding author.
